# Characterization of Cognitive Function in Survivors of Diffuse Gliomas Using Morphometric Correlation Networks

**DOI:** 10.3390/tomography8030116

**Published:** 2022-05-26

**Authors:** Chencai Wang, Nicholas S. Cho, Kathleen Van Dyk, Sabah Islam, Catalina Raymond, Justin Choi, Noriko Salamon, Whitney B. Pope, Albert Lai, Timothy F. Cloughesy, Phioanh L. Nghiemphu, Benjamin M. Ellingson

**Affiliations:** 1 UCLA Brain Tumor Imaging Laboratory (BTIL), Center for Computer Vision and Imaging Biomarkers, David Geffen School of Medicine, University of California Los Angeles, Los Angeles, CA 90024, USA; chencaiwang@mednet.ucla.edu (C.W.); nscho@mednet.ucla.edu (N.S.C.); sabahislam99@gmail.com (S.I.); craymond@mednet.ucla.edu (C.R.); 2 Department of Radiological Sciences, David Geffen School of Medicine, University of California Los Angeles, Los Angeles, CA 90024, USA; nsalamon@mednet.ucla.edu (N.S.); wpope@mednet.ucla.edu (W.B.P.); 3 Medical Scientist Training Program, David Geffen School of Medicine, University of California Los Angeles, Los Angeles, CA 90095, USA; 4 Department of Bioengineering, Henry Samueli School of Engineering and Applied Science, University of California Los Angeles, Los Angeles, CA 90095, USA; 5 Department of Psychiatry and Biobehavioral Sciences, David Geffen School of Medicine, Semel Institute, University of California Los Angeles, Los Angeles, CA 90095, USA; kvandyk@mednet.ucla.edu; 6 Department of Psychology, University of California Los Angeles, Los Angeles, CA 90095, USA; 7 Department of Neurology, David Geffen School of Medicine, University of California Los Angeles, Los Angeles, CA 90095, USA; justin.yoon.choi@gmail.com (J.C.); albertlai@mednet.ucla.edu (A.L.); tcloughesy@mednet.ucla.edu (T.F.C.); pnghiemphu@mednet.ucla.edu (P.L.N.); 8 Department of Neurosurgery, University of California Los Angeles, Los Angeles, CA 90095, USA

**Keywords:** diffuse gliomas, quality of life, cognitive function, magnetic resonance imaging, morphometric correlation network, cortical thickness

## Abstract

This pilot study investigates structural alterations and their relationships with cognitive function in survivors of diffuse gliomas. Twenty-four survivors of diffuse gliomas (mean age 44.5 ± 11.5), from whom high-resolution T1-weighted images, neuropsychological tests, and self-report questionnaires were obtained, were analyzed. Patients were grouped by degree of cognitive impairment, and interregional correlations of cortical thickness were computed to generate morphometric correlation networks (MCNs). The results show that the cortical thickness of the right insula (*R*^2^ = 0.3025, *p* = 0.0054) was negatively associated with time since the last treatment, and the cortical thickness of the left superior temporal gyrus (*R*^2^ = 0.2839, *p* = 0.0107) was positively associated with cognitive performance. Multiple cortical regions in the default mode, salience, and language networks were identified as predominant nodes in the MCNs of survivors of diffuse gliomas. Compared to cognitively impaired patients, cognitively non-impaired patients tended to have higher network stability in network nodes removal analysis, especially when the fraction of removed nodes (among 66 nodes in total) exceeded 55%. These findings suggest that structural networks are altered in survivors of diffuse gliomas and that their cortical structures may also be adapting to support cognitive function during survivorship.

## 1. Introduction

While surgical resection of tumors may extend patient survival, post-operative morbidity can significantly diminish patients’ quality of life [1]. For example, cognitive deficits in language, memory, attention, and executive function have been reported by patients following surgery and/or aggressive treatment [2,3]. The magnitude of severity of cognitive dysfunction varies from mild symptoms to deficits comparable to those seen in dementia syndromes [3,4]. Comprehensive neuropsychological assessment, self-reported cognitive symptoms, and daily functioning assessments have been used to characterize cognitive impairment and inform rehabilitation strategies [4,5,6,7,8]. In addition to these methods, there has been interest in utilizing magnetic resonance imaging (MRI) techniques to obtain biomarkers associated with cognitive function. For example, previous studies in patients with diffuse gliomas using resting-state functional MRI (rs-fMRI) have shown an association between altered functional network connectivity in the default mode network, executive control network, hand motor network, and salience network and in memory, attention, motor, and cognitive performance [9,10,11]. Moreover, the brain may undergo reorganization as a result of tumor resection; a previous study observed decreased connectivity in the sensorimotor network immediately following surgery, which eventually increased to pre-operative levels 3 months post-surgery [12]. However, there is limited knowledge of the structural alterations and their relationships with cognitive function in survivors of diffuse gliomas.

Morphometric correlation network (MCN) analysis has been used to investigate large-scale structural alterations by identifying patterns in cortical morphometry, such as gray matter thickness and volume [13,14]. This integrative approach uses cortical morphometry correlations between different brain regions to identify patterns in MCNs. From these MCNs, more sophisticated analyses can be performed, including quantification of network efficiency and stability, characterized by small-world architecture and robustness, respectively, and identification of critical nodes of each network [15,16]. MCN analysis has revealed specific patterns of structural alterations in patients with multiple sclerosis, schizophrenia, Alzheimer’s disease, breast cancer, and epilepsy [17,18,19,20,21]. However, to our knowledge, MCNs have not yet been explored in survivors of diffuse gliomas, which may be due to the presence of tumor resection cavities that can impact MCN analysis.

Therefore, in this exploratory pilot study, we characterize the MCNs of survivors of diffuse gliomas after accounting for tumor resection cavities and explore the associations between cortical thickness and cognitive function. We hypothesize that there will be significant differences between the MCNs of cognitively non-impaired and impaired glioma survivors and that alterations in cortical thickness would be associated with self-reported cognitive function, self-reported daily function, and time elapsed since surgery.

## 2. Materials and Methods

### 2.1. Patient Population

From 2018 to 2019, patients with primary brain tumors, seen at our institution’s neuro-oncology clinic for routine follow-up visits, were enrolled in this study with the following inclusion criteria: (1) age over 18 years old, (2) glioma grade II-IV via pathological diagnosis, (3) were not on any active therapies (surgery, radiation, chemotherapy, and/or targeted therapy) for at least 6 months, (4) no evidence of tumor progression at the time of the study, and (5) were able to undergo an entire-brain MRI imaging procedure, to follow verbal directions, and to complete written questionnaires and cognitive testing in English. There were no exclusion or inclusion criteria based on previous types of treatments. After providing their written informed consent, patients completed a T1-weighted MRI, a neuropsychological test battery, and a self-reported questionnaire within a seven-day span. All analyses were done in compliance with the Health Insurance Portability and Accountability Act (HIPAA), and our Institutional Review Board approved all aspects of the current study (IRB#11–001876; Medical IRB Committee #3).

### 2.2. T1-Weighted MR Image Acquisition and Post-Processing

To evaluate alterations in cortical thickness, high-resolution 1 mm 3-dimensional (3D) T1-weighted structural MRIs were acquired on a 3T MR scanner (Siemens Prisma; Siemens Healthcare, Erlangen, Germany) using a 3D magnetization-prepared rapid gradient-echo (MPRAGE) sequence in either the coronal, sagittal, or axial orientation, with a repetition time (TR) of 2300 to 2500 ms, a minimum echo time (TE), an inversion time (TI) of 900 to 945 ms, a flip angle of 9° to 15°, FOV = 240 × 320 mm and matrix size of 240 × 320, slice thickness = 1 mm.

**Figure 1:**
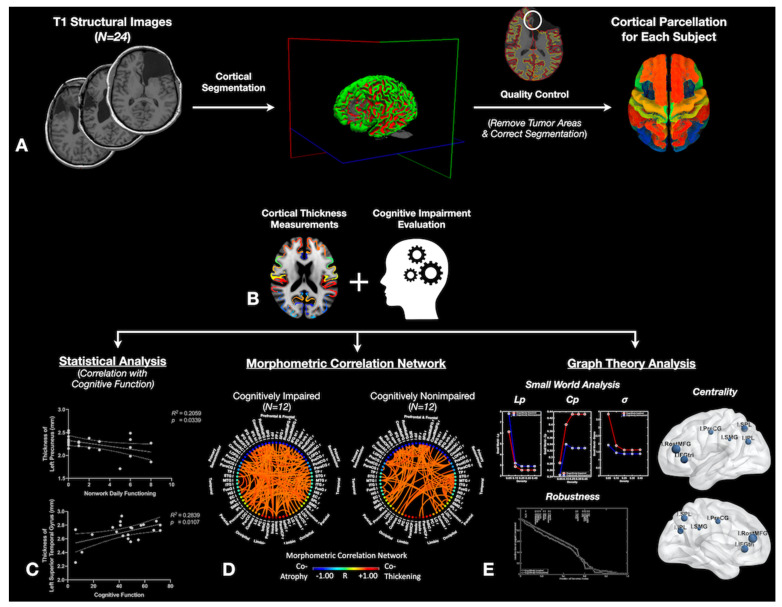
Image processing and analysis pipeline. (**A**) T1 structural images were processed using Freesurfer, followed by quality control to ensure no cortical segmentation errors. (**B**) After extracting the cortical thickness of each participant, this information was combined with cognitive impairment information. From there, (**C**) statistical analyses, including Pearson’s correlation analysis, were performed to find associations between regional cortical thickness and functional outcomes. (**D**) Cortical thickness was also used to construct the morphometric correlation network (MCN). (**E**) In-house Matlab scripts incorporating the Graph Theory GLM tool to analyze the MCN properties, including small-world analysis, centrality, and robustness. Full presentation of the findings in (**C**–**E**) are presented in the Results section.

T1-weighted images were processed using FreeSurfer (version 7.1; https://surfer.nmr.mgh.harvard.edu/fswiki (accessed on 20 March 2019).; Laboratory for Computational Neuroimaging, Athinoula A. Martinos Center for Biomedical Imaging, Charlestown, MA, USA) [22,23,24,25]. Following the standard FreeSurfer cortical reconstruction and post-processing using the recon-all command, quality control was performed to verify accurate cortical segmentations. If there were errors in cortical segmentation, manual editing, including adding control points for white matter identification and removing the skull and dura, was performed to ensure the resection cavity and other potential issues were not included in the cortical segmentation (Figure 1A). Processed brain surfaces were smoothed using a full-width half-maximum (FWHM) of 10 mm and then registered to standard space. Age, which is known to have a linear relationship with cortical thickness [25,26,27], was included as a covariate in all analyses.

### 2.3. Cognitive and Functional Outcomes

Cognitive impairment was determined based on patients’ performance and combined with imaging measurements (Figure 1B) using the following neuropsychological test battery: the Hopkins Verbal Learning Test—Revised [28]; the Brief Visuospatial Memory Test—Revised [29]; the Trail-Making Test [30,31]; the Wechsler Adult Intelligence Scale-IV Coding and Digit Span subtests; the Conner’s Continuous Performance Test-II [32]; the Golden Stroop Test [33]; the Verbal Fluency (FAS and animal naming) Test [34]; the Boston Naming Test [35]; and the Rey-Osterrieth Complex Figure—a visuospatial test [36]. Raw scores were converted into standard Z-scores using published normative data. We defined cognitive impairment according to the International Cognition and Cancer Task Force (ICCTF) guidelines, accounting for the number of test scores in the battery [37,38]. Patients were categorized as “cognitively impaired” if they had at least two test scores ≤−2 Z; otherwise, patients were categorized as “cognitively non-impaired”.

The Functional Assessment of Cancer Therapy-Cognitive Function (FACT-Cog) version 3 [8] measures subjective cognitive functioning by asking participants to rate several types of cognition-related concerns on a five-point Likert scale. Although the FACT-Cog yields four subscores, we focused on the Perceived Cognitive Impairment (PCI) subscore as the generally preferred outcome from this instrument [39]. The PCI scale has a range from 0 to 72, with higher values indicating better functioning.

Daily functioning was measured using the Work Productivity and Activity Impairment (WPAI) instrument [40]. Since work status is often affected in many brain tumor patients, we specifically selected the ability measure of non-work functioning, the scale of which has a range from 1 to 10, with higher values indicating poorer functioning. The scores were then transformed into percentiles.

### 2.4. Region of Interest Analysis

Following image post-processing, the cortex was divided into 66 regions of interest (ROIs) (33 for each hemisphere) based on the Desikan–Killiany cortical atlas template. The average cortical thickness was extracted within each ROI for every patient. Unpaired t-tests were conducted to identify significant differences in cortical thickness between cognitively non-impaired and impaired patients. Pearson’s correlation analyses were conducted to identify ROIs showing a significant association between cortical thickness and FACT-Cog PCI, WPAI Ability, and the time elapsed since surgery. The level of significance was set at *p* < 0.05, with age included as a covariate (Figure 1C).

### 2.5. Morphometric Correlation Network Construction

MCNs can be described as an interregional symmetric correlation matrix [41], where the magnitude of correlation coefficients represents the weight of the specific grid regions in the MCN. The current study used cortical thickness to calculate the interregional correlation and thus construct the MCN. To do this, cortical thickness measurements were first obtained for each cortical region (defined in the Desikan–Killiany cortical atlas template) of each individual participant, and then multiple linear regression was implemented to remove the effects of age (*p* < 0.05). Interregional Pearson’s correlation coefficients were evaluated between the age-corrected cortical thickness of every pair of anatomical ROIs cross-sectionally across individuals using a two-sided level of significance (*p* < 0.05) to construct MCNs (Figure 1D).

### 2.6. MCN Statistical Analysis

**Figure 2:**
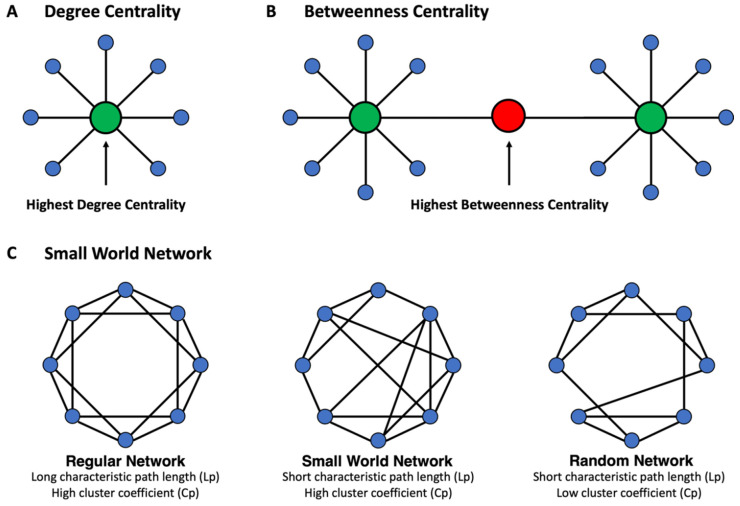
Basic concept of network properties and measures used in the current study. (**A**) Degree centrality is defined as the number of directly connected nodes. (**B**) Betweenness centrality measures the node’s role in acting as a bridge between separate clusters by computing the ratio of all shortest paths in the network that contains a given node. (**C**) The regular network (left) has a long characteristic path length (Lp) and high clustering coefficient (Cp), while the random network (right) has a short characteristic path length (Lp) and low clustering coefficient (Cp). The small-world network (middle) has an intermediate balance between regular and random networks, with a short path length (Lp) but high clustering coefficient (Cp).

In-house Matlab scripts incorporating the Graph Theory GLM tool (www.nitrc.org/projects/metalab_gtg) (accessed on 20 March 2019), were used to calculate and analyze the MCN properties as well as to compute region-weighted network centrality metrics (Figure 1E). In graph theory and network analyses, indicators of centrality identify the predominant nodes within a graph or a whole network [16,42]. The current study mainly focused on two indices of centrality metrics: (1) degree centrality, which reflects the number of neighbors a brain region interacts with (Figure 2A, see detailed information and mathematical formula in Supplementary Methods); and (2) betweenness centrality, which reflects the ability of a region to influence information flow between two other regions (Figure 2B, see detailed information and mathematical formula in Supplementary Methods). Nodes with both high degree centrality and high betweenness centrality are considered to be predominant nodes [16,42]. Additionally, small-world architecture, which ensures both the efficiency of specialization and the integration of distributed networks [16,42,43], was characterized by computing the cluster coefficient (Cp) and the characteristic path length (Lp) (Figure 2C, see detailed information and mathematical formula in Supplementary Methods) [15].

The MCN stability for both cognitively non-impaired and impaired groups was also evaluated using network robustness, which is characterized by the extent of tolerance against target attacks (Figure 1E). Specifically, target attacks refer to the removal of network nodes in decreasing order of their betweenness centrality. Each time after removing a node, the relative size of the sub-network was calculated by taking the ratio of the number of mutually reachable nodes in the largest remaining sub-network divided by 66, which was the number of nodes in the original network [21]. Sub-networks that more closely preserve the size of the original network are said to have more network robustness. A non-parametric permutation test with 1000 repetitions was applied to determine the significant difference in MCN stability between cognitively non-impaired and impaired patients [44]. To do this, the cortical thickness data of each participant was randomly reassigned to one of the two patient groups in each randomization procedure, followed by measuring the difference in the network robustness between the two groups. This generated a permutation distribution of differences under the null hypothesis. The 95th percentile (level of significance, *p* < 0.05) of the resulting permutation distribution was used to obtain the critical values for a two-tailed test of the null hypothesis.

## 3. Results

### 3.1. Patient Data

The study cohort included sixteen males and eight females, with a mean age of 44.5 years (range 26 to 69), as outlined in Table 1. All patients were right-handed. Of the 24 patients in our study, 12 were categorized as cognitively non-impaired and 12 as cognitively impaired based on the International Cognition and Cancer Task Force (ICCTF) guidelines. All patients successfully completed the Work Productivity and Activity Impairment (WPAI) Test for non-work daily functioning and the Functional Assessment of Cancer Therapy-Cognitive Function (FACT-Cog) for self-reported cognitive impairment. The mean FACT-Cog PCI score suggested significant cognitive impairment across all survivors [45], and the mean WPAI Ability score indicated relatively low functional impairment, though both had a wide range. Patient performance is summarized in Table 2.

### 3.2. Morphometric Correlation Networks in Cognitively Impaired and Non-Impaired Patients

**Figure 3:**
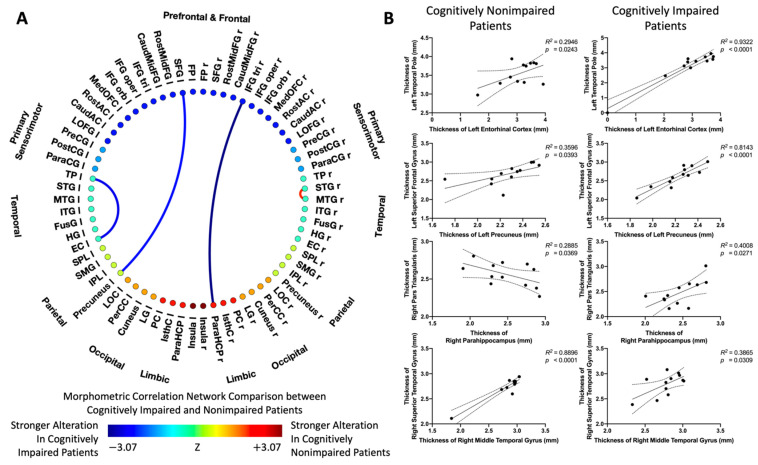
(**A**) Comparison of morphometric correlation networks (MCNs) between cognitively impaired and non-impaired patients. Colors denote values of the Z-statistic; yellow–red represents a stronger alteration in cognitively non-impaired patients; cyan–blue denotes stronger alteration in cognitively impaired patients. (**B**) Representative interregional correlations in cognitively non-impaired and impaired patients.

Compared to the cognitively impaired patients (Figure 3), cognitively non-impaired patients had stronger positive interregional correlations between the right superior temporal gyrus (r STG) and the right middle temporal gyrus (r MTG). However, cognitively impaired patients were found to show stronger positive interregional correlations between the left entorhinal cortex (l EC) and the left temporal pole (l TP), between the left precuneus and the left superior frontal gyrus (l SFG), and the right parahippocampus (l PaHC) and the right pars triangularis (r IFGtri).

**Figure 4:**
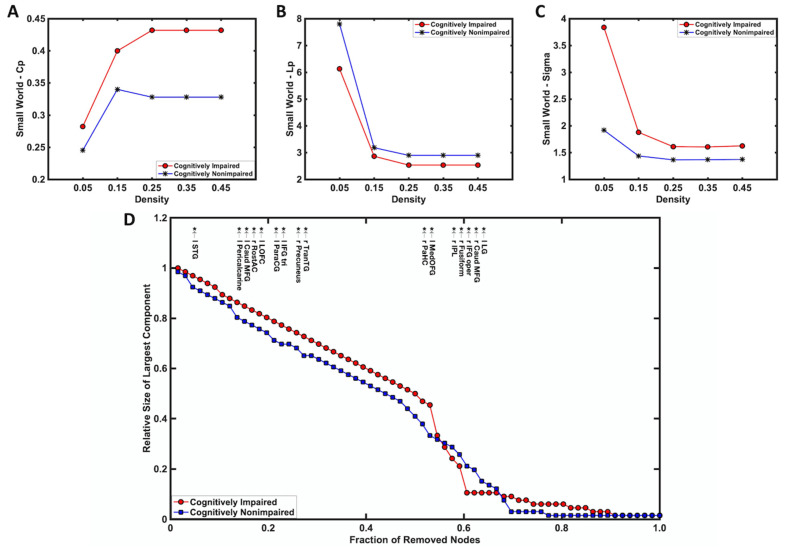
The small-world organization of brain networks, including (**A**) cluster coefficient (Cp), (**B**) characteristic path length (Lp), (**C**) small-world index (Sigma), and (**D**) topological robustness of morphometric correlation network, were quantified in cognitively impaired (red circle) and non-impaired (blue square) patients.

The small-world organization of brain networks was quantified for both cognitively non-impaired and impaired patients (Figure 4). Compared to cognitively impaired patients, non-impaired patients had a smaller cluster coefficient (Cp; Figure 4A) and a longer characteristic path length (Lp; Figure 4B) over a wide range of density, resulting in the cognitively impaired patients having a larger small-world index (Sigma; Figure 4C). In order to assess the network robustness of both cognitively non-impaired and impaired patients in response to the removal of targeted nodes, changes in the size of the remaining largest component of the network were plotted as a function of fractions of removing nodes in descending order of nodal betweenness centrality (Figure 4D). Compared to the cognitively non-impaired group, the cognitively impaired group showed more network tolerance when removing a small fraction of targeted nodes, but robustness was significantly reduced (*p* < 0.05) when the fraction of removed nodes exceeded 0.55.

**Figure 5:**
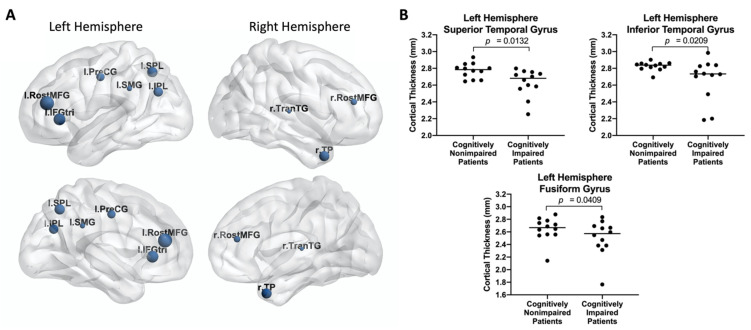
(**A**) The distribution of predominant nodes in both cognitively impaired and non-impaired patients. (**B**) Representative comparisons of cortical thickness between cognitively non-impaired and impaired patients.

Cognitively non-impaired and impaired patients shared some common predominant nodes with high centrality (Figure 5A), located at the left precentral gyrus (l PreCG), left supramarginal gyrus (l SMG), left superior parietal lobule (l SPL), left inferior parietal lobule (l IPL), left pars triangularis (l IFGtri), right transverse temporal gyrus (r TranTG), right temporal pole (r TP), and bilateral rostral middle frontal gyrus (RostMFG). When compared to cognitively impaired patients (Figure 5B), the cognitively non-impaired patients were observed to have thicker gray matter in the left superior temporal gyrus (l STG, *p* = 0.0132), left inferior temporal gyrus (l ITG, *p* = 0.0132), and the left fusiform gyrus (*p* = 0.0409).

### 3.3. Morphometric Correlation Network Associations with Neuropsychological Assessments across All Survivors of Diffuse Gliomas

**Figure 6:**
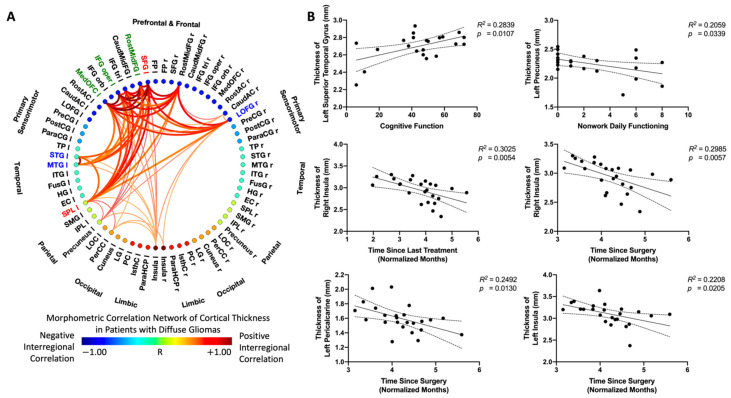
(**A**) Analysis of morphometric correlation networks (MCNs) of cortical thickness in patients with diffuse gliomas. Red–yellow denotes positive interregional correlation, while blue–light blue denotes negative interregional correlation. Green highlighted labels indicate nodes with high degree centrality, blue highlighted labels indicate nodes with high betweenness centrality, and red highlighted labels indicate nodes with both high degree and betweenness centrality. (**B**) Representative correlations between cortical thickness and cognitive function, between cortical thickness and non-work daily functioning, and cortical thickness and time since surgery in patients with diffuse gliomas.

The MCNs of cortical thickness across all survivors of diffuse gliomas identified the left superior parietal lobule and the left superior frontal gyrus as predominant nodes with both high degree centrality and high betweenness centrality (Figure 6A). Additionally, the left rostral middle frontal gyrus, left pars opercularis, and left medial orbitofrontal gyrus demonstrated high degree centrality, while the left superior temporal gyrus, left middle temporal gyrus, and right lateral orbitofrontal gyrus demonstrated high betweenness centrality.

In general, positive correlations were observed among predominant regions (Figure 6A). Additionally, the cortical thickness of the left superior temporal gyrus was positively associated with cognition, as evaluated by the FACT-Cog score (*R*^2^ = 0.2839, *p* = 0.0107), and the cortical thickness of the left precuneus was negatively associated with non-work daily functioning, as evaluated by WPAI Ability score (*R*^2^ = 0.2059, *p* = 0.0339) (Figure 6B). Furthermore, a negative association between cortical thickness and the elapsed time since last treatment was observed in the right insula (*R*^2^ = 0.3025, *p* = 0.0054), and negative associations between cortical thickness and the elapsed time since surgery were observed in the right insula (*R*^2^ = 0.2985, *p* = 0.0057), left insula (*R*^2^ = 0.2208, *p* = 0.0205), and left pericalcarine (*R*^2^ = 0.2492, *p* = 0.0130).

## 4. Discussion

In this cross-sectional study, the associations between cortical thickness and cognitive measures in survivors of diffuse gliomas and the differences in the MCNs of cognitively impaired and non-impaired diffuse glioma survivors were explored. The primary findings support our hypothesis that there exist widespread structural differences in survivors of diffuse gliomas. These findings are also consistent with previous reports of neurocognitive impairment [3,45], widespread functional and structural abnormalities [9,46], and large-scale morphological atrophy in glioma patients before tumor resection [17,47], along with post-operative cortical reorganization that is associated with better cognitive outcomes [48,49,50,51].

### 4.1. Morphometric Correlation Networks Reveal Large-Scale Cortical Alteration

Recent studies have demonstrated that cerebral networks can be constructed to characterize the disrupted coordination of brain morphology in many neurodegenerative diseases and psychiatric conditions, such as Alzheimer’s disease, epilepsy, and schizophrenia [17,18,19,20,52]. In the current study, we constructed MCNs of cortical thickness and demonstrated that both cognitively impaired and non-impaired glioma survivors showed increased positive correlations between the left entorhinal cortex and the left temporal pole, as well as between the left precuneus and left superior frontal gyrus. These interregional cortical thickness correlations were found to be significantly stronger in cognitively impaired patients and are consistent with morphological abnormalities previously observed in patients with mild impairment [53,54].

Additionally, the thicknesses of the right pars triangularis and the right parahippocampus were found to be negatively correlated in cognitively non-impaired glioma survivors but positively correlated in cognitively impaired glioma survivors. The right pars triangularis is involved with the semantic processing of language [55], while the parahippocampal region is well-known for its role in normal memory functioning, including verbal learning memory [56]. The fact that correlations between those two regions were reversed in non-impaired and impaired patients may suggest the existence of compensatory cortical reorganization following glioma resection that improves cognitive function for non-impaired survivors, as similarly demonstrated in previous studies [47,57].

The interregional correlation between the right middle temporal gyrus and the right superior temporal gyrus was also found to be positive in both cognitively impaired and non-impaired glioma survivors, but the correlation was significantly stronger in those with a better cognitive status. Furthermore, the left fusiform gyrus and the left superior and inferior temporal gyri were demonstrated to be thicker in non-impaired patients than in cognitively impaired patients. The observed patterns of alteration in the temporal lobe may also be explained by compensatory mechanisms in cognitively impaired and non-impaired survivors. The temporal lobe is involved in multiple complex functions, such as sensory processing, memory formation, language, and social recognition. While cognitive recovery has been reported after glioma resection [58], several studies have also shown that a decline in neurocognitive function occurs in post-operative patients whose resected gliomas were in the temporal lobe [59,60,61]. It has been suggested that cognitive function is more likely to become impaired as a result of damage caused by brain tumor resection in regions specifically relating to cognitive function across multiple cognitive tasks [50]. Thus, in the present study, a positive correlation of cortical thickness within the temporal lobes might suggest a possible co-preservation of cortical thickness that promotes cognitive stability in cognitively non-impaired patients but a possible co-atrophy of these regions in cognitively impaired patients due to the vulnerability of neurological decline following tumor resection. This speculation is further supported by the positive correlation observed between higher FACT-Cog scores and the thickness of the left superior temporal gyrus.

The network measurements derived from graph theory indicated small-world architecture (σ > 1) in the MCNs of both cognitively non-impaired and impaired survivors. Compared to cognitively impaired survivors, cognitively non-impaired survivors demonstrated a smaller cluster coefficient but a larger characteristic path length across a wide range of network densities, suggesting a disrupted brain network with lower efficiency and weaker local specialization. When examining network stability against targeted node removal, although the MCNs of cognitively non-impaired glioma survivors were found to be weaker at first, there was increased robustness compared to cognitively impaired glioma survivors when the fraction of removed nodes was over 0.55. Thus, we speculate that the improved cognitive status in non-impaired patients after the tumor resection may be due to cortical reorganization to non-traditional brain regions that may consequently reduce network robustness but also increase the stability of this network against the continuous removal of targeted nodes. Cortical structures have been demonstrated to reorganize in patients with a brain tumor as a result of the presence of the lesion itself or from surgical resection [12,62]. The findings in the present study may suggest that relatively stable networks can be reorganized in cognitively non-impaired patients; these networks can withstand more severe neurological deterioration than the networks in cognitively impaired patients.

### 4.2. Cortical Thickness Alterations Associated with Cognitive and Functional Outcomes

Regions with high degree centrality and between centrality are considered predominant nodes in MCNs. Predominant nodes generally demonstrate strong relationships with other regions in the MCN and are likely to be the most altered areas in glioma survivors. Among all seeding regions within the MCN, the left superior parietal lobule and the left superior frontal gyrus were identified as predominant nodes. Additionally, the left rostral middle frontal gyrus, left pars opercularis, and left medial orbitofrontal gyrus demonstrated high degree centrality, while the left superior temporal gyrus, left middle temporal gyrus, and right lateral orbitofrontal gyrus demonstrated high betweenness centrality. Changes to those anatomical regions, as identified using different imaging modalities, were not only frequently associated with structural atrophy and functional abnormalities in glioma patients [17,47,54] but also proved to be possible imaging biomarkers for monitoring disease progression from mild cognitive impairment to Alzheimer’s disease [54].

Note that some predominant nodes also demonstrate significant cortical thickness alterations associated with cognitive and functional outcomes. For example, a positive correlation was observed between higher FACT-Cog scores and the thickness of the left superior temporal gyrus, which was consistent with the observation revealed by the MCN comparisons between cognitively non-impaired and impaired survivors. Additionally, worsened non-work daily functioning was found to be associated with decreased cortical thickness in the left precuneus. The left superior temporal gyrus is known for its critical role in the language network [63], and the precuneus is known for its role in the default mode network and shows more activation in reward attainment [64]. As a result, these findings may demonstrate that the cortical thickness of predominant nodes may serve as biomarkers of cognitive function in glioma survivors.

Furthermore, time since surgery was negatively correlated with the cortical thickness of the left and right insula and the left primary visual cortex, and time since last treatment was negatively correlated with the cortical thickness of only the left insula. The insula is a major site of sensory, motor, and emotional integration in the brain, and it is considered a hub of the salience network [65]. Gliomas have been previously shown to cause altered functional connectivity of the insula, including the visual network [66]. Interestingly, the results correlating cortical thickness to time since surgery and time since last treatment were similar but not identical. All but one patient in our cohort received chemoradiation following surgical resection of the tumor. Chemoradiation itself has been suggested to cause alterations in cortical thickness in patients with brain tumors [67]. As a result, the observed associations between cortical thickness alterations and greater elapsed time since last treatment and surgery in the current study may suggest that treatments can have a long-term impact on brain structural features. However, it is important to note that these differences in cortical thickness relationships with time since last treatment and time since surgery should be interpreted with caution because this study involves cross-sectional analyses, not longitudinal analyses, so no conclusions can be made on the direct impacts of these treatments on cortical thickness. Nevertheless, the brains of glioma survivors may be undergoing competing adaptive mechanisms related to treatment effects and supporting cognitive function, which could potentially serve as indicators of successful rehabilitation during survivorship.

### 4.3. Limitation and Further Consideration

Several limitations in the current study should be noted. First, although quality control and manual editing (adding control points for white matter identification and removing the skull and dura) were implemented to correct errors in automated cortical segmentation, a more robust method to account for the tumor resection cavity and other potential issues in the cortical segmentation of diffuse glioma survivors can benefit the cortical parcellation and extraction of cortical morphometry, such as gray matter thickness and volume. Additionally, the MCN for patients with diffuse gliomas was generated using cortical thickness; however, a more comprehensive model using cortical volume would further benefit the characterization of morphological measures. Therefore, going forward, we plan to develop a novel technique to quantify the cortical volume following brain tumor resection. Lastly, the findings of the current study were based on cross-sectional data obtained from a small population of glioma patients with a broad range of diagnoses and treatments, lesion locations, as well as varying severities and nature of cognitive impairment. It is possible that the findings in the present study may not be fully generalizable to all survivors of diffuse gliomas because of the limited sample size. For example, 15 out of the 24 patients in our study cohort had tumors that involved the frontal cortex, and so, the present findings may be impacted by this limited tumor location distribution. However, associations involving ROIs of the frontal cortex were still able to be discovered, so the present findings likely represent the general brain tumor patient population. Furthermore, there was still a wide distribution of cognitive performance scores across the study cohort despite the small sample size. Nevertheless, the findings between cognitively impaired and non-impaired patients may also be impacted by heterogeneity in tumor location between the two groups, which is why much of the discussions on the present findings and potential compensatory mechanisms are prefaced as speculations. A larger cohort, follow-up testing, and longitudinal data are necessary to further study the observed morphometric correlations and the associations between structural alterations and cognitive impairment in relation to cortical reorganization. Longitudinal data would be particularly valuable to better elucidate how MCNs may be changing over time, with changing cognitive performance scores, while controlling for MCN alterations that reflect the presence and location of the tumor within each patient.

## 5. Conclusions

In the current cross-sectional study, we characterized MCN patterns in diffuse glioma survivors and the correlation between cortical thickness and cognitive measures. Our results demonstrate that large-scale cortical alterations are associated with cognition, non-work daily functioning, and time points during treatment. Altered structural patterns may be useful as possible clinical biomarkers in evaluating treatment outcomes in survivors of diffuse gliomas.

## Figures and Tables

**Table 1:** Clinical data of patients.

ID	Age	Sex	Tumor Location	Tumor Grade	IDH1/2 Status	Rad	Chemo	Years Since Surgery	Years Since Last Treatment	Cognitively Impaired
1	38	M	R FC	WHO II	Mutant	Y	Y	6.43	4.75	N
2	38	M	L FC	WHO III	Mutant	Y	Y	1.95	1.73	N
3	42	M	R PC	WHO III	Mutant	Y	Y	6.00	4.42	N
4	39	M	L FC	WHO III	Mutant	Y	Y	5.07	3.81	Y
5	50	F	L FC	WHO III	Unknown	Y	Y	8.88	6.68	N
6	46	F	R FC	WHO IV	Mutant	Y	Y	7.18	5.98	N
7	31	M	R FPC	WHO II	Mutant	Y	Y	3.13	1.69	Y
8	32	M	R TC	WHO III	Mutant	Y	Y	5.00	3.84	Y
9	41	M	R FC	WHO III	Mutant	Y	Y	7.42	5.94	N
10	45	M	L FC	WHO III	Mutant	Y	Y	3.89	2.77	N
11	62	M	R FC	WHO III	Mutant	Y	Y	5.15	4.98	Y
12	57	M	L FC	WHO IV	Mutant	Y	Y	9.00	7.45	Y
13	42	F	L OC	WHO IV	Wild Type	Y	Y	8.26	6.40	Y
14	61	F	R FC	WHO III	Mutant	Y	Y	2.36	1.23	Y
15	22	M	R FTC	WHO III	Mutant	Y	Y	3.86	2.54	N
16	29	M	L TC	WHO II	Mutant	N	N	4.49	4.49	N
17	70	M	R FC	WHO IV	Wild Type	Y	Y	4.57	2.42	Y
18	48	M	R PC	WHO IV	Mutant	Y	Y	10.99	8.17	N
19	45	F	L PC	WHO III	Unknown	Y	Y	14.67	12.37	N
20	46	M	L TC	WHO II	Mutant	Y	Y	6.73	5.43	Y
21	52	F	R FC	WHO II	Mutant	Y	Y	2.51	0.70	Y
22	28	F	L TC	WHO II	Mutant	Y	Y	2.91	1.35	Y
23	38	M	L TC	WHO II	Mutant	Y	Y	5.83	0.60	Y
24	60	F	L FC	WHO III	Unknown	Y	Y	22.39	21.63	N

Y = yes; N = no; M = male; F = female; R = right; L = left; Rad = radiation; Chemo = chemotherapy. FC = frontal cortex; PC = parietal cortex; TC = temporal cortex; OC = occipital cortex; FPC = frontoparietal cortex; FTC = frontotemporal cortex. WHO = World Health Organization; IDH1/2 = isocitrate dehydrogenase −1/2.

**Table 2:** Patients’ performance on neuropsychological assessments.

Test	Performance
WPAI Non-Work Ability Impairment (Mean ± SD) [Min, Max]	26% ± 29% [0, 80%]
Functional Assessment for Cancer Therapy-Cognitive Function, Perceived Cognitive Impairment (Mean ± SD) [Min, Max]	43.7 ± 19.6 [6, 72]

SD = standard deviation.

## Data Availability

The data presented in this study are available upon request from the corresponding author.

## Supplementary Materials

The following supporting information can be downloaded at: https://www.mdpi.com/article/10.3390/tomography8030116/s1. See Refs [68,69,70,71], Supplementary Methods: Characterization of Cognitive Function in Survivors of Diffuse Gliomas using Morphometric Correlation Networks.

## References

[1] Buckner J.C., Shaw E.G., Pugh S.L., Chakravarti A., Gilbert M.R., Barger G.R., Coons S., Ricci P., Bullard D., Brown P.D. (2016). Radiation plus Procarbazine, CCNU, and Vincristine in Low-Grade Glioma. N. Engl. J. Med..

[2] Gehrke A.K., Baisley M.C., Sonck A.L., Wronski S.L., Feuerstein M. (2013). Neurocognitive deficits following primary brain tumor treatment: Systematic review of a decade of comparative studies. J. Neurooncol..

[3] Aaronson N.K., Taphoorn M.J., Heimans J.J., Postma T.J., Gundy C.M., Beute G.N., Slotman B.J., Klein M. (2011). Compromised health-related quality of life in patients with low-grade glioma. J. Clin. Oncol..

[4] Mackworth N., Fobair P., Prados M.D. (1992). Quality of life self-reports from 200 brain tumor patients: Comparisons with Karnofsky performance scores. J. Neurooncol..

[5] Feuerstein M., Hansen J.A., Calvio L.C., Johnson L., Ronquillo J.G. (2007). Work productivity in brain tumor survivors. J. Occup. Env. Med..

[6] Dwan T.M., Ownsworth T., Chambers S., Walker D.G., Shum D.H. (2015). Neuropsychological assessment of individuals with brain tumor: Comparison of approaches used in the classification of impairment. Front. Oncol..

[7] Noll K.R., Fardell J.E. (2015). Commentary: “Neuropsychological Assessment of Individuals with Brain Tumor: Comparison of Approaches Used in the Classification of Impairment”. Front. Oncol..

[8] Wagner L.I., Sweet J., Butt Z., Lai J.S., Cella D. (2009). Measuring Patient Self-Reported Cognitive Function: Development of the Functional Assessment of Cancer Therapy–Cognitive Function Instrument. J. Support. Oncol..

[9] Maesawa S., Bagarinao E., Fujii M., Futamura M., Motomura K., Watanabe H., Mori D., Sobue G., Wakabayashi T. (2015). Evaluation of resting state networks in patients with gliomas: Connectivity changes in the unaffected side and its relation to cognitive function. PLoS ONE.

[10] Harris R.J., Bookheimer S.Y., Cloughesy T.F., Kim H.J., Pope W.B., Lai A., Nghiemphu P.L., Liau L.M., Ellingson B.M. (2014). Altered functional connectivity of the default mode network in diffuse gliomas measured with pseudo-resting state fMRI. J. Neurooncol..

[11] Mallela A.N., Peck K.K., Petrovich-Brennan N.M., Zhang Z., Lou W., Holodny A.I. (2016). Altered Resting-State Functional Connectivity in the Hand Motor Network in Glioma Patients. Brain Connect..

[12] Vassal M., Charroud C., Deverdun J., Le Bars E., Molino F., Bonnetblanc F., Boyer A., Dutta A., Herbet G., Moritz-Gasser S. (2017). Recovery of functional connectivity of the sensorimotor network after surgery for diffuse low-grade gliomas involving the supplementary motor area. J. Neurosurg..

[13] Yang J.J., Kwon H., Lee J.M. (2016). Complementary Characteristics of Correlation Patterns in Morphometric Correlation Networks of Cortical Thickness, Surface Area, and Gray Matter Volume. Sci. Rep..

[14] Yates D. (2012). Neurodegenerative networking. Nat. Rev. Neurosci..

[15] Watts D.J., Strogatz S.H. (1998). Collective dynamics of ‘small-world’ networks. Nature.

[16] Rubinov M., Sporns O. (2010). Complex network measures of brain connectivity: Uses and interpretations. Neuroimage.

[17] Bouwen B.L.J., Pieterman K.J., Smits M., Dirven C.M.F., Gao Z., Vincent A. (2018). The Impacts of Tumor and Tumor Associated Epilepsy on Subcortical Brain Structures and Long Distance Connectivity in Patients with Low Grade Glioma. Front. Neurol..

[18] Raj A., Powell F. (2018). Models of Network Spread and Network Degeneration in Brain Disorders. Biol. Psychiatry Cogn. Neurosci. Neuroimaging.

[19] Manuello J., Nani A., Premi E., Borroni B., Costa T., Tatu K., Liloia D., Duca S., Cauda F. (2017). The Pathoconnectivity Profile of Alzheimer’s Disease: A Morphometric Coalteration Network Analysis. Front. Neurol..

[20] Seeley W.W., Crawford R.K., Zhou J., Miller B.L., Greicius M.D. (2009). Neurodegenerative diseases target large-scale human brain networks. Neuron.

[21] Bernhardt B.C., Chen Z., He Y., Evans A.C., Bernasconi N. (2011). Graph-theoretical analysis reveals disrupted small-world organization of cortical thickness correlation networks in temporal lobe epilepsy. Cereb. Cortex.

[22] Dale A.M., Fischl B., Sereno M.I. (1999). Cortical surface-based analysis. I. Segmentation and surface reconstruction. Neuroimage.

[23] Fischl B., Dale A.M. (2000). Measuring the thickness of the human cerebral cortex from magnetic resonance images. Proc. Natl. Acad. Sci. USA.

[24] Fischl B., Sereno M.I., Dale A.M. (1999). Cortical surface-based analysis. II: Inflation, flattening, and a surface-based coordinate system. Neuroimage.

[25] Salat D.H., Buckner R.L., Snyder A.Z., Greve D.N., Desikan R.S., Busa E., Morris J.C., Dale A.M., Fischl B. (2004). Thinning of the cerebral cortex in aging. Cereb. Cortex.

[26] Lemaitre H., Goldman A.L., Sambataro F., Verchinski B.A., Meyer-Lindenberg A., Weinberger D.R., Mattay V.S. (2012). Normal age-related brain morphometric changes: Nonuniformity across cortical thickness, surface area and gray matter volume?. Neurobiol. Aging.

[27] Sowell E.R., Peterson B.S., Kan E., Woods R.P., Yoshii J., Bansal R., Xu D., Zhu H., Thompson P.M., Toga A.W. (2007). Sex differences in cortical thickness mapped in 176 healthy individuals between 7 and 87 years of age. Cereb. Cortex.

[28] Benedict R.H., Schretlen D., Groninger L., Brandt J. (2001). Hopkins Verbal Learning Test—Revised.

[29] Benedict R.H., Schretlen D., Groninger L., Dobraski M., Shpritz B. (1997). Brief. Visuospatial Memory Test—Revised.

[30] Heaton R.K., Taylor M.J., Grant I. (2004). Revised Comprehensive Norms for an Expanded Halstead Reitan Battery: Demographically Adjusted Neuropsychological Norms for African American and Caucasian Adults.

[31] Reitan R.M., Wolfson D. (1985). The Halstead-Reitan Neuropsychological Test Battery: Theory and Interpretattion.

[32] Conners C.K., Staff M. (2000). Conners’ Continuous Performance Test. II (CPT II V. 5).

[33] Golden C.J., Freshwater S.M. (2002). The Stroop Color and Word Test: A Manual for Clinical and Experimental Uses.

[34] Strauss E., Sherman E.M., Spreen O. (2006). A Compendium of Neuropsychological Tests: Administration, Norms, and Commentary.

[35] Kaplan E., Goodglass H., Weintraub S. (2001). The Boston Naming Test.

[36] Meyers J.E., Meyers K.R. (1995). Rey Complex Figure Test and Recognition Trial.

[37] Ingraham L.J., Aiken C.B. (1996). An empirical approach to determining criteria for abnormality in test batteries with multiple measures. Neuropsychology.

[38] Wefel J.S., Vardy J., Ahles T., Schagen S.B. (2011). International Cognition and Cancer Task Force recommendations to harmonise studies of cognitive function in patients with cancer. Lancet Oncol..

[39] System F.M. FACIT Questionnaires: FACT-Cog Scoring and Interpretation Materials. https://www.facit.org/FACITOrg/Questionnaires.

[40] Reilly M.C., Zbrozek A.S., Dukes E.M. (1993). The validity and reproducibility of a work productivity and activity impairment instrument. Pharmacoeconomics.

[41] He Y., Chen Z., Evans A. (2008). Structural Insights into Aberrant Topological Patterns of Large-Scale Cortical Networks in Alzheimer’s Disease. J. Neurosci..

[42] Sporns O. (2018). Graph theory methods: Applications in brain networks. Dialogues Clin. Neurosci..

[43] Sporns O. (2011). The human connectome: A complex network. Ann. N. Y. Acad. Sci..

[44] Bullmore E.T., Suckling J., Overmeyer S., Rabe-Hesketh S., Taylor E., Brammer M.J. (1999). Global, voxel, and cluster tests, by theory and permutation, for a difference between two groups of structural MR images of the brain. IEEE Trans. Med. Imaging.

[45] Dyk K.V., Crespi C.M., Petersen L., Ganz P.A. (2019). Identifying Cancer-Related Cognitive Impairment Using the FACT-Cog Perceived Cognitive Impairment. JNCI Cancer Spectr..

[46] Liu D., Liu Y., Hu X., Hu G., Yang K., Xiao C., Hu J., Li Z., Zou Y., Chen J. (2020). Alterations of white matter integrity associated with cognitive deficits in patients with glioma. Brain Behav..

[47] Xu J., Elazab A., Liang J., Jia F., Zheng H., Wang W., Wang L., Hu Q. (2017). Cortical and Subcortical Structural Plasticity Associated with the Glioma Volumes in Patients with Cerebral Gliomas Revealed by Surface-Based Morphometry. Front. Neurol..

[48] Magill S.T., Han S.J., Li J., Berger M.S. (2018). Resection of primary motor cortex tumors: Feasibility and surgical outcomes. J. Neurosurg..

[49] Lin J., Lv X., Niu M., Liu L., Chen J., Xie F., Zhong M., Qiu S., Li L., Huang R. (2017). Radiation-induced abnormal cortical thickness in patients with nasopharyngeal carcinoma after radiotherapy. Neuroimage Clin..

[50] Niki C., Kumada T., Maruyama T., Tamura M., Kawamata T., Muragaki Y. (2020). Primary Cognitive Factors Impaired after Glioma Surgery and Associated Brain Regions. Behav. Neurol..

[51] Yoshii Y., Tominaga D., Sugimoto K., Tsuchida Y., Hyodo A., Yonaha H., Kushi S. (2008). Cognitive function of patients with brain tumor in pre- and postoperative stage. Surg. Neurol..

[52] Bullmore E.T., Woodruff P.W., Wright I.C., Rabe-Hesketh S., Howard R.J., Shuriquie N., Murray R.M. (1998). Does dysplasia cause anatomical dysconnectivity in schizophrenia?. Schizophr. Res..

[53] Long X., Jiang C., Zhang L. (2018). Morphological Biomarker Differentiating MCI Converters from Nonconverters: Longitudinal Evidence Based on Hemispheric Asymmetry. Behav. Neurol..

[54] Li S., Yuan X., Pu F., Li D., Fan Y., Wu L., Chao W., Chen N., He Y., Han Y. (2014). Abnormal changes of multidimensional surface features using multivariate pattern classification in amnestic mild cognitive impairment patients. J. Neurosci..

[55] Wildgruber D., Ackermann H., Kreifelts B., Ethofer T. (2006). Cerebral processing of linguistic and emotional prosody: fMRI studies. Prog. Brain Res..

[56] von Cramon D.Y., Hebel N., Schuri U. (1988). Verbal memory and learning in unilateral posterior cerebral infarction. A report on 30 cases. Brain.

[57] Li Q., Dong J.W., Del Ferraro G., Petrovich Brennan N., Peck K.K., Tabar V., Makse H.A., Holodny A.I. (2019). Functional Translocation of Broca’s Area in a Low-Grade Left Frontal Glioma: Graph Theory Reveals the Novel, Adaptive Network Connectivity. Front. Neurol..

[58] Teixidor P., Gatignol P., Leroy M., Masuet-Aumatell C., Capelle L., Duffau H. (2007). Assessment of verbal working memory before and after surgery for low-grade glioma. J. Neurooncol..

[59] Noll K.R., Weinberg J.S., Ziu M., Wefel J.S. (2016). Verbal Learning Processes in Patients with Glioma of the Left and Right Temporal Lobes. Arch. Clin. Neuropsychol..

[60] Noll K.R., Ziu M., Weinberg J.S., Wefel J.S. (2016). Neurocognitive functioning in patients with glioma of the left and right temporal lobes. J. Neurooncol..

[61] Sherman E.M., Wiebe S., Fay-McClymont T.B., Tellez-Zenteno J., Metcalfe A., Hernandez-Ronquillo L., Hader W.J., Jette N. (2011). Neuropsychological outcomes after epilepsy surgery: Systematic review and pooled estimates. Epilepsia.

[62] Cho N.S., Peck K.K., Zhang Z., Holodny A.I. (2018). Paradoxical Activation in the Cerebellum During Language fMRI in Patients with Brain Tumors: Possible Explanations Based on Neurovascular Uncoupling and Functional Reorganization. Cerebellum.

[63] Chang E.F., Rieger J.W., Johnson K., Berger M.S., Barbaro N.M., Knight R.T. (2010). Categorical speech representation in human superior temporal gyrus. Nat. Neurosci..

[64] Bradley K.A., Stern E.R., Alonso C.M., Xie H., Kim-Schulze S., Gabbay V. (2019). Relationships between neural activation during a reward task and peripheral cytokine levels in youth with diverse psychiatric symptoms. Brain Behav. Immun..

[65] Cauda F., D’Agata F., Sacco K., Duca S., Geminiani G., Vercelli A. (2011). Functional connectivity of the insula in the resting brain. Neuroimage.

[66] Almairac F., Deverdun J., Cochereau J., Coget A., Lemaitre A.L., Moritz-Gasser S., Duffau H., Herbet G. (2021). Homotopic redistribution of functional connectivity in insula-centered diffuse low-grade glioma. Neuroimage Clin..

[67] Seibert T.M., Karunamuni R., Kaifi S., Burkeen J., Connor M., Krishnan A.P., White N.S., Farid N., Bartsch H., Murzin V. (2017). Cerebral Cortex Regions Selectively Vulnerable to Radiation Dose-Dependent Atrophy. Int. J. Radiat. Oncol. Biol. Phys..

[68] Liu J., Li M., Pan Y., Lan W., Zheng R.Q., Wu F.X., Wang J.X. (2017). Complex Brain Network Analysis and Its Applications to Brain Disorders: A Survey. Complexity.

[69] Anthonissen J.M. (1971). The Rush in a Directed Graph.

[70] Freeman L.C. (1977). A Set of Measures of Centrality Based on Betweenness. Sociometry.

[71] Humphries M.D., Gurney K., Prescott T.J. (2006). The brainstem reticular formation is a small-world, not scale-free, network. Proc. Biol. Sci..

