# Supplementary material for: Characterization of Cognitive Function in Survivors of Diffuse Gliomas Using Morphometric Correlation Networks
Source: Tomography. 2022 May 26;8(3):1437–52. doi: 10.3390/tomography8030116 (PMC9229761; doi:10.3390/tomography8030116)
Supplement: Supplementary file 1 [file tomography-08-00116-s001.zip › tomography-1699114-supplementary.pdf]

Supplementary Methods

1. Morphometric Correlation Network Analysis

In-house Matlab scripts incorporating the Graph Theory GLM tool (www.nitrc.org/projects/metalab_gtg) were used to calculate and analyze the Morphometric Correlation Network (MCN) properties as well as to compute the region-weighted network centrality metrics.

1.1. Node Degree

Node degree of a brain network is often denoted as k, it is a simple count of the number of connections or edges the node has to all other nodes [1,2]. In general, the higher the degree of a node, the more nodes it connects to and the more central it is in the brain network. In a binary undirected network, the degree ki of a node i is defined as

$$ki= j \neq iaij$$

where $aij= 1$ if there is connection or edge between node i and node j; otherwise $aij= 0$.

1.2. Cluster Coefficient

The cluster coefficient of a node measures the degree to which nodes in a graph tend to cluster together. The higher the clustering coefficient, the more locally efficient the networks is [2]. In a binary undirected network, the clustering coefficient $C(i)$ of a node i is equal to the ratio of the number of the actual connections or edges between adjacent nodes to the number of all connections or edges; that could possibly exist between them, which is,

$$Ci = 2tiki(ki - 1)$$

Where $ti= 12i \leftrightarrow j \leftrightarrow haijaihajh$ denotes the number of triangles around a node i within a network.

The clustering coefficient of the network is simply calculated by averaging clustering coefficient of all nodes in the network, which is

$$C = 1NiC(i) = 1Ni2tiki(ki - 1)$$

1.3. Shortest Path Length

The shortest path plays a major role in the communication within a brain network, and it is a very important measure to describe the internal structure of the brain network [1,2]. The shortest

path can help facilitate the fast transmission of information and reduce brain consumption. In a binary undirected network, a path between node i and node j with the minimum number of connections or edges is defined as the shortest path, and its length lij cab be denoted as

$$lij = ast \in li \leftrightarrow jast$$

where $li \leftrightarrow j$ is the shortest path between node i and node j.

The characteristic path length L of a network is calculated by averaging the shortest path length between all possible pairs of nodes in the network

$$L = 1Nili$$

The characteristic path length is a measure of functional integration, which is the ability to rapidly combine pieces of specialized information from distributed brain regions [2].

1.4. Centrality

The centrality is to measure the importance of nodes in a brain network. Nodes with high centrality are considered to be brain hubs. In the current study of MCN, two measures of centrality are used: degree centrality, and betweenness centrality.

1.4.1. Degree Centrality

Degree centrality is the simplest measure of centrality, which uses the node degree to describe the importance of the node in the brain network [1,2]. In brain network analysis, the degree centrality of a brain region measures the direct impact of the brain region on neighbors. The degree centrality $Cd(i)$ of a node i has the same definition as the node degree:

$$Cdi = ki = j \neq iaij$$

1.4.2. Betweenness Centrality

Betweenness centrality quantifies the number of times that a node acts as a bridge along the shortest path between two other nodes [1,2]. In brain network analysis, the betweenness centrality of a brain region reflects the ability of a region to influence information flow between two other regions [3,4]. The betweenness centrality $Cb(i)$ of a node i is defined as the proportion of shortest paths between nodes j and h that pass through i:

$$Cbi = 2(N-1)(N-2)j \neq h \neq inhj(i)nhj$$

where $nhj(i)$ is the number of shortest paths between nodes j and h that pass through i, nhj is the number of all shortest paths between nodes j and h, and $(N-1)(N-2)/2$ is the number of node pairs that exclude node i.

1.5. Small-World Network

The small-world networks have both high clustering characteristics (compatible with segregated processing), and shorter shortest path lengths (compatible with integrated processing) [1,2]. If a brain network is found to have small-world property, it will ensure both efficiency of specialization and integration of distributed brain regions. Three measures are used to characterize if a network is a small-world network:

$$\gamma = CCrand \qquad \lambda = LLrand \qquad \sigma = \gamma\lambda$$

where $Crand$ and $Lrand$ are the average clustering coefficient and characteristic path length of M matched random networks that preserve the same number of nodes, edges, and degree distribution as the real network, γ and λ are the normalized clustering coefficient and normalized characteristic path length of the network, and σ is the small-world index of the network [5]. A network with small-world property needs to meet two conditions: $\gamma \gg 1$ and $\lambda \approx 1$, resulting in the small-world index $\sigma > 1$.

REFERENCES

1. Liu, J.; Li, M.; Pan, Y.; Lan, W.; Zheng, R.Q.; Wu, F.X.; Wang, J.X. Complex Brain Network Analysis and Its Applications to Brain Disorders: A Survey. *Complexity* **2017**, doi:Unsp 836274110.1155/2017/8362741.
2. Rubinov, M.; Sporns, O. Complex network measures of brain connectivity: uses and interpretations. *Neuroimage* **2010**, *52*, 1059-1069, doi:10.1016/j.neuroimage.2009.10.003.
3. Anthonissen, J.M. *The rush in a directed graph*; Stichting Mathematisch Centrum: Mathematische Besliskunde, 1971.
4. Freeman, L.C. *A set of measures of centrality based on betweenness*; Sociometry: 1977; Volume 40.
5. Humphries, M.D.; Gurney, K.; Prescott, T.J. The brainstem reticular formation is a small-world, not scale-free, network. *Proc Biol Sci* **2006**, *273*, 503-511, doi:10.1098/rspb.2005.3354.
